# Editorial: Metabolic profiles of autistic and typically developing children

**DOI:** 10.3389/fpsyt.2022.1005521

**Published:** 2022-08-15

**Authors:** Roberto Palumbi

**Affiliations:** Department of Basic Medical Sciences, Neurosciences and Sensory Organs, School of Medicine, University of Bari Aldo Moro, Bari, Italy

**Keywords:** autism (ASD), neurobiology, metabolic profile analysis, metabolic analyses, neurodevelopmental disorders

Autism Spectrum Disorder (ASD) is a common neurodevelopment disorder, characterized by an impairment in social communication/interaction and restricted interests/stereotyped behaviors. So far, a strong effort has been made in order to shed a light on the neurobiology of this condition, and current research is focused on specific Research Topics related to some intriguing etiopathogenic hypotheses.

The main aim of this Research Topic was to present brand-new data collected by investigations on potential metabolic ASD peripheral biomarkers that might support new neurobiological bases of the disorder (neuroimmunological, metabolic, and genetic), in the perspective to discover new tools for the diagnosis and new potential therapeutic interventions.

Four research teams gave their great contributions to this Research Topic. Qi et al. carried out a study investigating the reliability of a new rat model for ASD. They repeatedly transplated the fecal extract sample from ASD patients into pregnant rats to develop a new rat model called oFMT. Interestingly, they found that oFMT rats showed typical autistic behaviors; moreover, oFMT microbiota resembles the gut microbiota alterations of ASD patients, but quite different from previous valproic acid rat model (oVPA) microbiota. In addition, the authors found a specific serological profile of oFMT, when compared with oVPA characterized by specific level of norepinephrine, 5-OT and gamma-aminobutyric acid. Overall, even if with some limitations, the authors demonstrated that autism oFMT might be used as a new potential model with a high validity and reliability.

Another interesting contribution that would be a great preliminary work for future studies was offered by the research of Yao et al. Based on the evidence that parents' health status directly impacts therapeutic outcome of ASD children, the authors found that the nitric oxide (NO) metabolites urine levels in ASD children parents (p-ASD) might be potential biomarkers for measuring their physical and mental stress levels, as they showed significantly different NO urine metabolites when compared with healthy non-ASD-parents adult. Xu et al. presented the data of a preliminary study aiming to identify a peculiar metabolic profile of autistic patients using plasma and urine metabolic analyses. Their results suggest that there is a metabolic alteration in children with ASD, particularly in taurine and hypotaurine metabolism, phenylalanine metabolism, and arginine and proline metabolism. Further and more comprehensive studies on targeted metabolic pathways would be useful to validate these preliminary results.

Lastly, based on the neuroimmunological hypothesis for ASD, Zhao et al. conducted an interesting meta-analysis of the studies investigating the peripheral blood cytokines profile in children with ASD. Growing evidence suggests that the immune system plays a key role in ASD neurobiology. Their systematic review showed that the levels of peripheral IL-6, IL-1β, IL-12p70, macrophage migration inhibitory factor (MIF), eotaxin-1, monocyte chemotactic protein-1 (MCP-1), IL-8, IL-7, IL-2, IL-12, tumor necrosis factor-α (TNF-α), IL-17, and IL-4 were abnormal cytokines in the peripheral blood of ASD patients compared with controls.

On the whole, the scientific contributions to this Research Topic support the evidence that autistic patients might present a peculiar metabolic profile, caused by an aberration of specific metabolic pathways or by an immune dysfunction. Considering these data, in the next future, further studies would support these results in the effort to establish valid peripheral biomarkers useful to support not only the diagnosis but also to provide new clues for innovating and effective targeted treatments.

## Author contributions

The author confirms being the sole contributor of this work and has approved it for publication.

## Conflict of interest

The author declares that the research was conducted in the absence of any commercial or financial relationships that could be construed as a potential conflict of interest.

## Publisher's note

All claims expressed in this article are solely those of the authors and do not necessarily represent those of their affiliated organizations, or those of the publisher, the editors and the reviewers. Any product that may be evaluated in this article, or claim that may be made by its manufacturer, is not guaranteed or endorsed by the publisher.

